# Genome-wide identification, characterization, and evolutionary analysis of the barley TALE gene family and its expression profiles in response to exogenous hormones

**DOI:** 10.3389/fpls.2024.1421702

**Published:** 2024-06-27

**Authors:** Tian-jiang Liao, Tao Huang, Hui-yan Xiong, Jie-cuo Duo, Jian-zhi Ma, Ming-yang Du, Rui-jun Duan

**Affiliations:** ^1^ College of Eco-environmental Engineering, Qinghai University, Xining, Qinghai, China; ^2^ College of Agriculture and Animal Husbandry, Qinghai University, Xining, Qinghai, China

**Keywords:** barley, TALE, gene family, KNOX, BELL, hormones treatment, growth and development

## Abstract

Three-amino-loop-extension (TALE) family belongs to the homeobox gene superfamily and occurs widely in plants, playing a crucial role in regulating their growth and development. Currently, genome-wide analysis of the TALE family has been completed in many plants. However, the systematic identification and hormone response analysis of the TALE gene family in barley are still lacking. In this study, 21 TALE candidate genes were identified in barley, which can be divided into KNOX and BELL subfamilies. Barley TALE members in the same subfamily of the phylogenetic tree have analogically conserved motifs and gene structures, and segmental duplications are largely responsible for the expansion of the HvTALE family. Analysis of TALE orthologous and homologous gene pairs indicated that the HvTALE family has mainly undergone purifying selective pressure. Through spatial structure simulation, HvKNOX5–HvKNOX6 and HvKNOX5–HvBELL11 complexes are all formed through hydrogen bonding sites on both the KNOX2 and homeodomain (HD) domains of HvKNOX5, which may be essential for protein interactions among the HvTALE family members. Expression pattern analyses reveal the potential involvement of most *HvTALE* genes in responses to exogenous hormones. These results will lay the foundation for regulation and function analyses of the barley TALE gene family in plant growth and development by hormone regulation.

## 1 Introduction

Homeobox genes encode a prodigious superfamily of transcription factors (TFs), which occur widely in animals, plants, and other eukaryotes. A typical homeobox domain comprises a triple helix structure of 60 amino acids, forming a ring structure in the first and second helix regions and forming a helix-turn-helix structure in the second and third helix regions (Billeter et al., 1993). The first plant homeobox gene, maize *Knotted1*, was reported by Vollbrecht et al. (1991). Three-amino-loop-extension (TALE) TFs with a non-classical homeobox domain comprising 63 amino acids, and three further residues (P-Y-P) inserted between the first and second helices, are referred to as the TALE gene family. Based on protein sequences and evolution, this family is divided into a knotted-like homeodomain (KNOX) subfamily, and a BEL1-like homeodomain (BELL) subfamily, which interacts between specific individuals (Hake et al., 2004; Hamant and Pautot, 2010). Selective targeting of KNOX–BELL heterodimer to the nucleus may be the purpose of interaction between BELL and KNOX proteins (Bhatt et al., 2004; Kim et al., 2013). KNOX proteins usually have four domains (KNOX1, KNOX2, ELK, and homeodomain). According to gene structural characteristics, the KNOX subfamily has been divided into three classes: KNOX I, II, and III. The KNOX III class lacks homeodomains and was only reported in dicotyledons (Hake et al., 2004). BELL subfamily proteins, which contain a MID (also known as POX) composed of BELL and SKY domains and a homeodomain (HD), were less well studied (Müller et al., 2001; Cole et al., 2006). BEL1-like proteins have not been systematically classified to date.

The TALE family members play a vital role in regulating plant growth and development (Lin et al., 2013) and maintaining organ morphology (Belles-Boix et al., 2006), signal transduction (Cnops et al., 2006), hormone regulation (Shani et al., 2006), tuber formation (Kondhare et al., 2019), and resistance to abiotic stress (Tao et al., 2018). The KNOX gene subfamily has been intensively studied. KNOX I genes are primarily expressed in meristems and have distinct roles (Hake et al., 2004; Hay and Tsiantis, 2010; Qi and Zheng, 2013). For instance, *Arabidopsis KNAT2* is expressed in the apical meristems, influences AGAMOUS (AG) ectopic expression in the carpel and ovule center, and causes the nucellar structure to homomorphically convert to a carpel-like structure (Pautot et al., 2001). The *KNAT1* mutation causes a decrease in *PIN2* expression levels in the root tip, enhances auxin accumulation in roots, and downregulates auxin transport in basal leaves. According to these findings, KNAT1 may adversely affect root tilt by regulating auxin transport (Qi and Zheng, 2013). KNOX II genes were expressed in a variety of tissues. Among these genes, *KNAT7* is important for secondary cell wall (SCW) biosynthesis and cell elongation in *Arabidopsis* (*AtKNAT7*), rice (*OsKNAT7*), cotton (*GhKNAT7-A03*), and poplar (*PoptrKNAT7*) (Li et al., 2012; Ma et al., 2019; Yu, 2019). To prevent more rhizobia infection and nodule development, *KNAT3/4/5*-like genes may activate the EFD/RR4 pathway, partially inhibiting cytokinin signaling, thereby regulating the nodular organ boundary and shape in *Medicago truncatula* (Vernié et al., 2008). KNOX III gene in *Arabidopsis* contains just one *KNATM*, which also affects leaf development and leaf polarity (Magnani and Hake, 2008). The functions of ATH1, BEL1, BLH2, BLH4, PNF/BLH8, and PNY/BLH9 in the *Arabidopsis* BELL subfamily have also been confirmed, but the roles of the other BELL-like genes remain unknown. For example, ATH1 is a flowering inhibitor that controls the expression level of FLOWERING LOCUS C (*FLC*) gene (Proveniers et al., 2007). BLH9 stimulates flowering when it interacts with BLH8, while it inhibits flowering when it interacts with ATH1 (Rutjens et al., 2009). One or more KNOX genes were regulated by *BLH2* and *BLH4* to prevent the growth of specific leaf subdomains (Kumar et al., 2007).

At present, genome-wide analysis of the TALE family has been completed in many plants such as *Arabidopsis thaliana* (Hamant and Pautot, 2010), *Populus trichocarpa* (Zhao et al., 2019), *Glycine max* (Wang et al., 2021), and *Triticum aestivum* (Han et al., 2022). Meanwhile, considering the importance of this family in the development of flowers and leaves in the Poaceae family, it is urgent and necessary to conduct research on the barley TALE gene family. However, the systematic identification and hormone response analysis of the TALE gene family in barley (*Hordeum vulgare*) are still lacking. Here, a whole genome-wide analysis of the TALE family in barley was fulfilled and identified. We also fulfilled a phylogenetic analysis and confirmed chromosome location, gene structure, and homology analysis, protein interactions, expression patterns of barley TALE genes (HvTALEs). This study will help us to better understand the roles of TALE family members in the growth and development of hormone regulation in barley. These studies depicted the characterization and diversity of HvTALE genes, revealing their roles in the growth and development of barley in the future.

## 2 Materials and methods

### 2.1 Identification and characteristics of TALE genes in barley

Barley protein sequences were downloaded from the Ensembl Plants database (http://plants.ensembl.org/index.Html) (Bolser et al., 2017). A Pfam domain (PF00046) was used to find members of the barley TALE family in the HMMER3 program (http://hmmer.org) using the hidden Markov model (HMM) searching method. To exclude genes lacking the conserved domain, the candidate barley TALE family members were submitted to the following databases for validation: Pfam (http://www.ebi.ac.uk/interpro/), SMART (https://smart.embl.de), and National Center for Biotechnology Information (NCBI) protein Batch CD-search (http://www.ncbi.nlm.nih.gov/Structure/bwrpsb/bwrpsb.cgi) (Yang et al., 2020; Mistry et al., 2021). Genes with HD and KNOX1 or KNOX2 domains were selected as KNOX subfamily members, and those with HD and POX domains were selected as BELL subfamily members, so barley TALE family members were identified in the barley genome. Then, amino acid (aa) numbers, isoelectric point (pI), and molecular weight (MW) of identified barley TALE proteins were predicted in ExPASy (https://web.expasy.org/compute_pi/) online tools (Artimo et al., 2012).

### 2.2 Phylogenetic analysis and classification of barley TALE proteins

Conserved protein sequences were extracted for multi-species phylogenetic analysis. Required barley TALE CDs and genomic DNA sequences were downloaded from the Ensembl Plants database. TALE protein sequences of *T. aestivum*, *G. max*, and *P. trichocarpa* were obtained from published papers (Zhao et al., 2019; Wang et al., 2021; Han et al., 2022). *Arabidopsis* protein sequences were obtained from TAIR (https://www.arabidopsis.org/), while TALE protein sequences of *Oryza sativa* and *Zea mays* were obtained from PlantTFDB v4.0 (http://planttfdb.gao-lab.org/) (Jin et al., 2017). Using the neighbor-joining (NJ) method, phylogenetic trees were constructed in MEGA7 software (https://www.megasoftware.net/) (Kumar et al., 2016) and EvolView 10 (https://evolgenius.info//evolview-v2/#login). Using Mapchart software, the location information of barley TALE superfamily members comes from the Ensembl Plants database (Voorrips, 2002), and its members were mapped to seven barley chromosomes.

### 2.3 Duplication and syntenic analyses of barley TALE genes

Using the MCScanX program, segmental and tandem duplications in the barley TALE genes were found (Gu et al., 2002; Kong et al., 2013; Wang et al., 2013). Duplicated gene pairs in the barley *H. vulgare* Morex v3 genome were prepared using Circos software (Krzywinski et al., 2009). Syntenic relationships between TALE superfamily members in barley and other species were identified using TBtools (Chen et al., 2020). Required barley TALE CDS and protein sequences were downloaded from the barley pan-genome, such as Akashinriki, Golden_Promise, and B1K-04–12 (Jayakodi et al., 2020), and the wild barley OUH602 genome (Liu et al., 2020). TALE orthologous gene pairs in barley were determined among Morex and four barley accessions by an alignment of full-length aa sequences. TBtools software was used to calculate non-synonymous/synonymous (Ks/Ka) ratios, and the Ka/Ks values were defined by three criteria: Ka/Ks < 1 (purifying selection), Ka/Ks = 1 (neutral selection), and Ka/Ks > 1 (positive selection) (Anisimova et al., 2001).

### 2.4 Protein motifs and gene structure characterization of barley TALE genes

GSDS (Gene Structure Display Server 2.0) (http://gsds.gao-lab.org/Gsds_help.php) was used to show gene structures (Hu et al., 2015). Conserved motifs in predicted barley TALE proteins were examined using the Multiple Expectation Maximization for Motif Elicitation (MEME) program (http://meme-suite.org/tools/meme) (Bailey et al., 2009) and were visualized by TBtools.

### 2.5 Identified *cis*-element analysis in the promoter region of TALE genes

The 2,000-bp region upstream of the start codon of barley TALE family members was used as the promoter sequence (Zhao et al., 2016). The promoter sequence was submitted to the PlantCARE database (http://bioinformatics.psb.ugent.be/webtools/plantcare/html/) to identify *cis*-elements (Lescot et al., 2002).

### 2.6 Interaction network of barley TALE genes and protein–protein interaction analysis

The Orthovenn2 site (https://www.genengnews.com/best-of-the-web/orthovenn2/) was used to map barley genes with *Arabidopsis* gene homology. Eight *Arabidopsis* TALE proteins representing eight barley TALE proteins were submitted to the STRING online server (https://string-db.org/) to predict protein–protein interactions. The barley TALE protein interaction network was visualized using Cytoscape (https://cytoscape.org/).

We use the FlyFish program with AlphaFold to realize automatic 3D structure modeling (Jumper et al., 2021). FlyFish is bioinformatics software that enables data analysis, task management, database operations, and more. We sought to write a 3D structure modeling analysis and data processing program in FlyFish, then run this program to automatically perform batch analysis in AlphaFold, and export results and 3D structures data. We selected reported interacting proteins as positive controls to identify the possibility of interaction between the two proteins of interest by MEGADOCK 4.0 docking software (Ohue et al., 2014).

### 2.7 Expression analysis of barley TALE genes in different tissues and under different exogenous hormone treatments

Transcriptional profiles were provided by the Leibniz Institute of Plant Genetics and Crop Plant Research (IPK) barley BARLEX server (https://pgrc.ipk-gatersleben.de/projects/barley/) in 14 tissues and at different development stages (Monat et al., 2019). The fragments per kilobase of transcript per million fragment mapped reads (FPKM) were used to quantify the levels of gene expression. A heatmap of gene expression data was constructed in TBtools software using log2-transformed mean FPKM values.

To explore expression patterns of barley TALE genes under hormone treatments, publicly available 57 RNA-seq samples were downloaded [nine RNA-seq samples of ABA and SA treatments from the Sequence Read Archive (SRA) database using transcripts per million (TPM) treats and 48 RNA-seq samples of ABA and MeJA treatments from Gene Expression Omnibus (GEO) BioProject in NCBI]. Accession numbers and sample information of RNA-seq are listed in **Supplementary Table 1**. Agilent Feature Extraction Software (v 9.1) was used for background subtraction and LOWESS normalization of GEO sample data. Two heatmaps for gene expression data were constructed in TBtools software using log2 (TPM+1) of SRA data and downloaded GEO sample data. All reads from these datasets were mapped to the Morex v3 genome (Mascher et al., 2021).

### 2.8 Plan materials, RNA isolation, and qRT-PCR

Barley variety ‘Morex’ was used for hormone treatments. After sterilization with 10% bleach for 5 min and rinsing thrice with deionized water, the seeds were put into Petri dishes to germinate in the dark for 48 h at 22°C. In growth chambers, sprouted seeds were grown under carefully regulated circumstances, including 55% relative humidity, 22°C, and a 16-h/8-h light/dark photoperiod with 20,000 lx of light intensity. Barley seedlings were exposed to 100 μM GA3, 100 μM ABA, and 100 μM 6-BA for hormone treatments at 12 d after germination. At 0 h, 3 h, 6 h, 9 h, 12 h, and 24 h, the leaves of barley seedlings were taken from all treatments and controls. TRIzol was used to extract RNA from barley leaves, and a FastKing RT Kit (TIANGEN, Beijing, China) was used to synthesize cDNA. Real-time quantitative PCR was carried out using PerfectStart™ GREEN qPCR SuperMix (SYBR Green I) (Transgen, Beijing, China). For every qRT-PCR analysis, the barley β-actin gene served as an internal reference. The 2^−ΔΔCT^ value was utilized for the analysis of gene expression data. The qRT-PCR-specific primers are listed in **Supplementary Table 2**.

## 3 Results

### 3.1 Identification and chromosomal distributions of barley TALE family members

We identified 21 HvTALE genes including nine KNOX genes (*HvKNOX1* to *HvKNOX9*) and 12 BELL genes (*HvBELL1* to *HvBELL12*) from *H. vulgare* Morex v3 genome, named according to their gene coordinate on the seven barley chromosomes (Han et al., 2022) (**Table 1**). These 21 HvTALE genes were unevenly distributed on the seven barley chromosomes (**Supplementary Figure 1**). Notably, Chr4H enclosed the most HvTALE genes (10), whereas Chr2, Chr3, and Chr6H had a single gene only.

**Table 1:** Physical and biochemical properties of TALE genes identified in barley.

Gene name	Gene ID	HvTALE subfamily	Chr.	Gene start (bp)	Gene end (bp)	Length (aa)	pI	Mw (kDa)	GRAVY	PSL
HvKNOX1	HORVU.MOREX.r3.1HG0016370.1	KNOX Class I	Chr1H	47,506,515	47,512,144	306	5.40	33.10021	−0.568	Nucleus
HvKNOX2	HORVU.MOREX.r3.2HG0154270.1	KNOX Class I	Chr2H	363,973,953	363,977,826	349	6.32	38.57452	−0.639	Nucleus, cytoplasm
HvKNOX3	HORVU.MOREX.r3.4HG0333990.1	KNOX Class I	Chr4H	7,173,641	7,185,231	321	5.65	35.78888	−0.822	Nucleus
HvKNOX4	HORVU.MOREX.r3.4HG0334040.1	KNOX Class I	Chr4H	7,401,532	7,405,517	353	8.74	39.2952	−0.736	Nucleus
HvKNOX5	HORVU.MOREX.r3.4HG0339120.1	KNOX Class I	Chr4H	24,234,352	24,242,112	364	6.31	40.34435	−0.599	Nucleus
HvKNOX6	HORVU.MOREX.r3.4HG0412370.1	KNOX Class II	Chr4H	594,364,765	594,369,936	315	5.92	34.84649	−0.505	Nucleus
HvKNOX7	HORVU.MOREX.r3.5HG0513530.1	KNOX Class I	Chr5H	535,228,694	535,235,584	420	8.09	46.42088	−0.471	Nucleus
HvKNOX8	HORVU.MOREX.r3.6HG0568300.1	KNOX Class II	Chr6H	111,189,013	111,194,443	297	5.95	32.41241	−0.563	Nucleus
HvKNOX9	HORVU.MOREX.r3.7HG0744200.1	KNOX Class II	Chr7H	613,060,146	613,064,327	298	5.85	32.84187	−0.614	Nucleus
HvBELL1	HORVU.MOREX.r3.1HG0052320.1	BEL1-like	Chr1H	348,794,735	348,800,106	596	6.61	63.63255	−0.515	Nucleus
HvBELL2	HORVU.MOREX.r3.1HG0073900.1	BEL1-like	Chr1H	464,310,250	464,314,963	580	8.34	62.68864	−0.621	Nucleus
HvBELL3	HORVU.MOREX.r3.3HG0302040.1	BEL1-like	Chr3H	547,804,242	547,808,949	611	7.91	66.03347	−0.601	Nucleus
HvBELL4	HORVU.MOREX.r3.4HG0334350.1	BEL1-like	Chr4H	8,338,174	8,339,591	342	5.44	37.49787	−0.556	Nucleus
HvBELL5	HORVU.MOREX.r3.4HG0334360.1	BEL1-like	Chr4H	8,722,968	8,726,333	636	5.39	70.23389	−0.688	Nucleus
HvBELL6	HORVU.MOREX.r3.4HG0339810.1	BEL1-like	Chr4H	27,555,371	27,565,963	809	6.29	85.47339	−0.569	Nucleus
HvBELL7	HORVU.MOREX.r3.4HG0350220.1	BEL1-like	Chr4H	98,313,718	98,318,816	666	5.88	69.90024	−0.507	Nucleus
HvBELL8	HORVU.MOREX.r3.4HG0402730.1	BEL1-like	Chr4H	561,849,331	561,852,756	589	5.80	62.84729	−0.566	Nucleus
HvBELL9	HORVU.MOREX.r3.4HG0412330.1	BEL1-like	Chr4H	594,228,348	594,233,452	622	6.03	66.86208	−0.594	Nucleus
HvBELL10	HORVU.MOREX.r3.5HG0420480.1	BEL1-like	Chr5H	3,415,223	3,417,664	649	5.80	72.14237	−0.73	Nucleus, cytoplasm
HvBELL11	HORVU.MOREX.r3.7HG0636950.1	BEL1-like	Chr7H	4,915,798	4,918,264	477	6.97	53.15032	−0.481	Nucleus
HvBELL12	HORVU.MOREX.r3.7HG0717740.1	BEL1-like	Chr7H	512,372,117	512,374,191	541	6.02	58.31130	−0.342	Nucleus

MW, molecular weight; pI, isoelectric point; aa, amino acid; PSL, predicted subcellular localization; GRAVY, grand average of hydropathicity.

Fundamental physical and biochemical characteristics of HvTALE family members were explored (**Table 1**), including protein length, isoelectric point, MW, grand average of hydropathicity, and subcellular localization. Protein sizes of HvTALE members varied from 297 aa (HvKNOX8) to 809 aa (HvBELL6), and corresponding MW ranged from 32.41241 to 85.47339 kDa (**Table 1**); pI values ranged from 5.40 (HvKNOX1) to 8.74 (HvKNOX4). Subcellular location predictions indicated that HvTALE proteins (19 members) occurred mainly in the nuclear region. Two HvTALE members (HvKNOX2 and HvBELL10) may occur in nuclear and cytoplasm regions. Gene CDS and protein sequences are presented in **Supplementary Table 3**, and HD sequences of barley HvTALE members are presented in **Supplementary Table 4**.

### 3.2 Phylogenetic analysis and classification of HvTALEs

**Figure 1:**
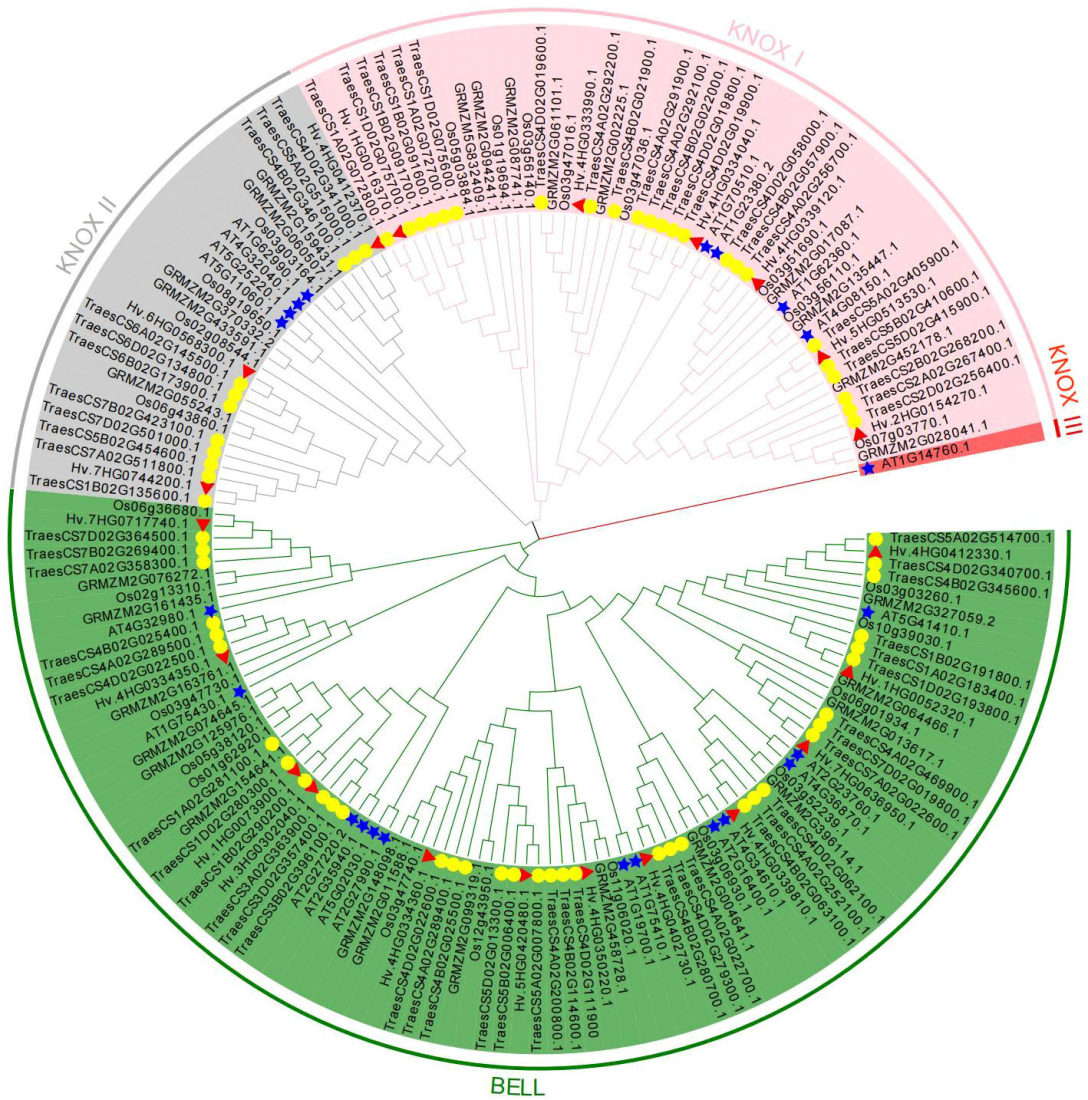
Phylogenetic analysis of HvTALE proteins. In total, 167 KNOX/BELL protein sequences from five species, including four monocotyledons (*Hordeum vulgare*, *Oryza sativa*, *Zea mays*, and *Triticum aestivum*) and one dicotyledon (*Arabidopsis thaliana*), were used to construct the unrooted neighbor-joining (NJ) tree. Different subclass genes are distinguished by color: KNOX I (pink), KNOX II (gray), KNOX III (red), and BELL (green). All barley TALE proteins are denoted by red triangles, *Arabidopsis* TALE proteins are represented by blue pentacles, and wheat TALE proteins are represented by yellow circles.

A phylogenetic tree was built using 167 TALE protein sequences from five species (*A. thaliana*, *H. vulgare*, *O. sativa*, *Z. mays*, and *T. aestivum*) to investigate the phylogeny and taxonomic relationships of TALE superfamily genes (**Figure 1**). Additionally, an unrooted evolutionary tree with just barley TALE proteins was also built (**Supplementary Figure 2**). Based on structural characteristics and the classification in *Arabidopsis* (**Supplementary Table 5**), the 21 HvTALE family members were classified into KNOX and BEL1-like subfamilies (**Table 1**). Nine members of the KNOX subfamily were further divided into six members of class KNOX I and three members of class KNOX II.

### 3.3 Gene structures and motif compositions of HvTALE members

**Figure 2:**
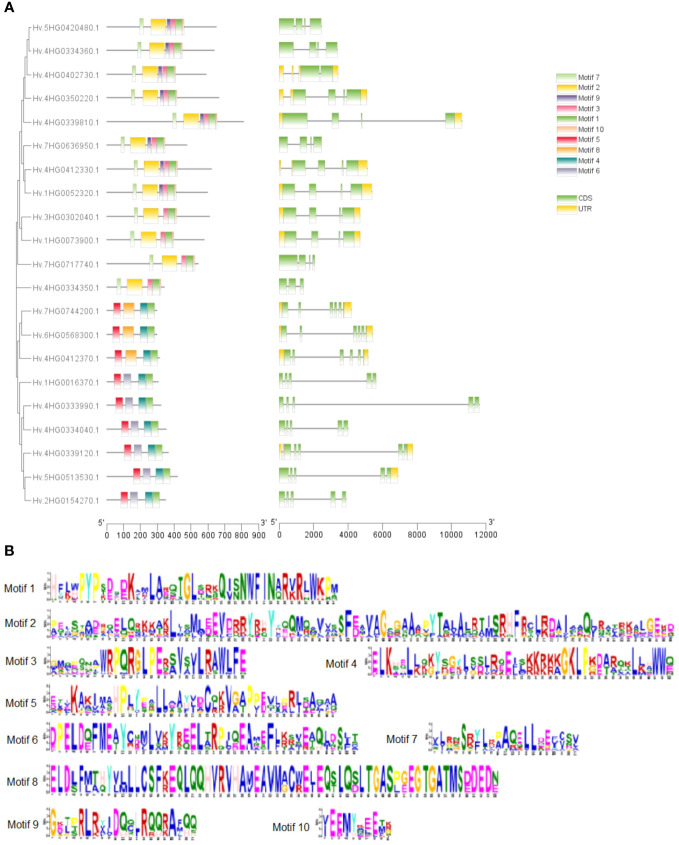
Gene structures and motif patterns of HvTALE members. **(A)** Gene structures and motif patterns of HvTALE genes. Colors: green boxes, exons; gray lines, introns; yellow boxes, untranslated 5′ and 3′ regions. Ten motifs are set by Multiple Expectation Maximization for Motif Elicitation (MEME) software. **(B)** Seq Logos of HvTALE members; 10 motifs are set by MEME software.

We investigated exon–intron patterns and conserved domain compositions of discovered HvTALE genes to gain a better understanding of possible relationships between the structure and function of HvTALE genes (**Figure 2A**). HvTALE genes displayed two to five exons (one HvTALE gene contains two exons, one HvTALE gene contains three exons, 10 HvTALE genes contain four exons, and nine HvTALE genes contain five exons). HvTALE genes in the same subfamily showed similar gene structures. Notably, except for HvBELL4 and HvBELL7, the BEL1-like subfamily genes had four exons, but the KNOX subfamily genes had five exons. Conserved domains of HvTALE members are depicted in **Figure 2A**. All HvTALE members contained HD domains, the BEL1-like subfamily members harbored POX domains, and KNOX1, KNOX2, and ELK domains occurred only in the KNOX subfamily. Significantly, the MEINOX domain, which mediates the formation of heterodimers between KNOX and BEL1-like proteins, is composed of the KNOX1 and KNOX2 domains (Bhatt et al., 2004; Kim et al., 2013).

To better understand conserved domain patterns, we used MEME online software to scan HvTALE gene motifs and set the motif quantity at 10. A drawing was constructed using the 10 scanned MEME-motifs in **Figure 2A**, and the MEME-motifs’ Sequence Logos are shown in **Figure 2B**. HvTALE members of the same subfamily also display similar motif compositions. Motifs 1–3 and 7–10 were present in most BEL1-like subfamily members, while Motifs 1, 4–6, and 8 were present in KNOX subfamily members. The KNOX subfamily showed different motif components across different clades. Class KNOX II members were linked to Motifs 1, 4, 5, and 8, while class KNOX I members contained Motifs 1 and 4–6. The BELL subfamily exhibited two types of motif components: four HvBELL members contained Motifs 1, 2, 5, 7, and 10, and others were associated with Motifs 1, 2, 5, 7, 9, and 10.

### 3.4 Expansion and evolutionary analyses of HvTALE genes

**Figure 3:**
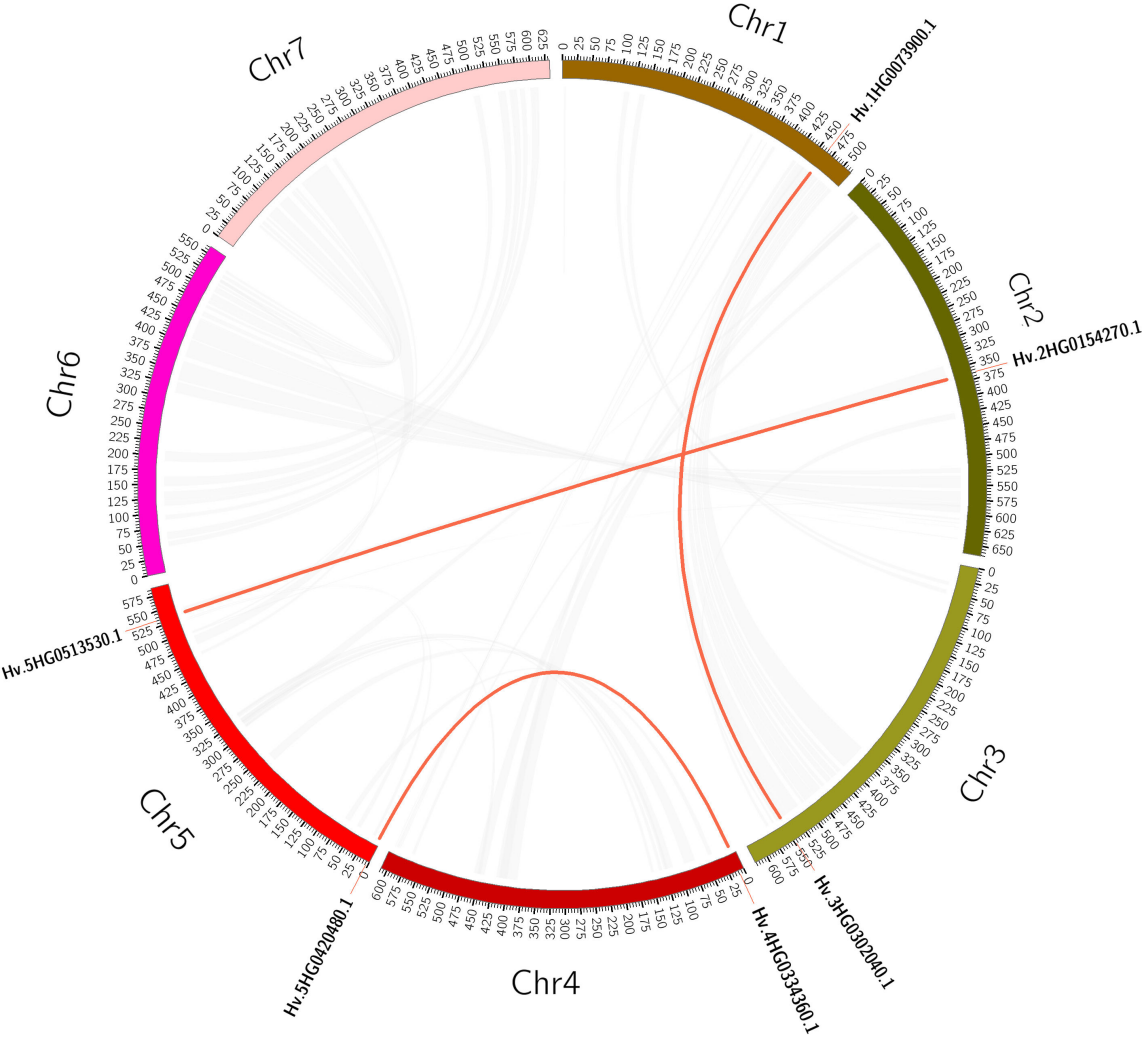
A circular figure showing the HvTALE genes’ collinearity. Duplicate HvTALE gene pairs are connected by red curves, and genome-wide collinear blocks are used as the background (gray).

Plant gene family expansions are thought to be primarily driven by tandem and segmental duplication events (Cannon et al., 2004). Tandem duplication events are defined as 200-kb chromosomal regions containing two or more genes (Han et al., 2022). We detected a tandem duplication event linked to two HvTALE genes (*HvBELL4*/*HvBELL5*) on Chr4H (**Supplementary Figure 1**). In contrast, segmental duplications result in a significant number of duplicated chromosomal blocks within genomes and frequently happen during chromosome rearrangement-related polyploidization events (Yu et al., 2005). In the barley genome, we found three segmental duplication events (**Supplementary Table 6A**, **Figure 3**). Compared with tandem duplications, the segmental duplications mainly drive the expansion of the HvTALE superfamily.

**Figure 4:**
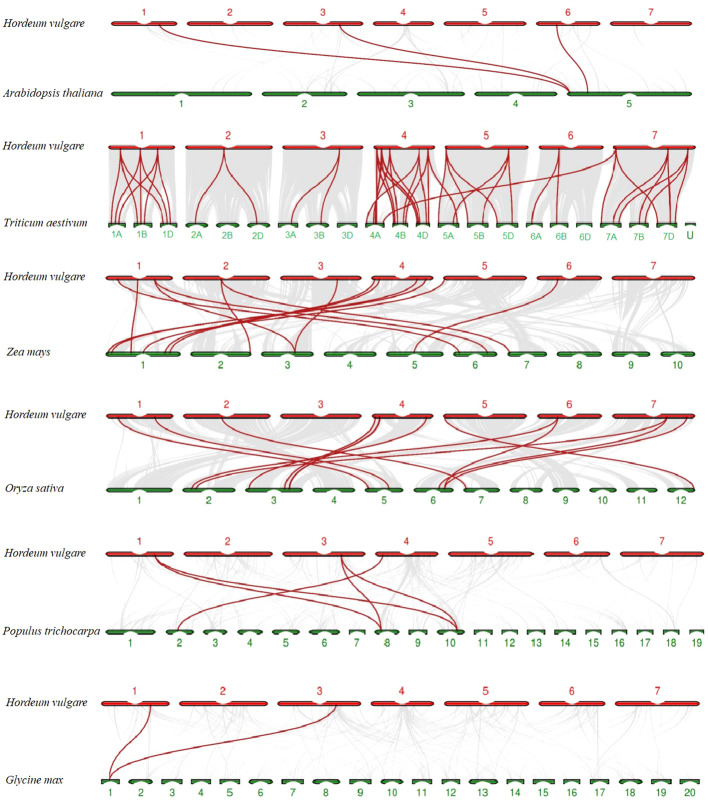
Syntenic relations of TALE members among barley and six plant species; 3, 2, 5, 15, 56, and 14 HvTALE orthologous genes were identified between barley and *Arabidopsis thaliana*, *Glycine max*, *Populus trichocarpa*, *Oryza sativa*, *Zea mays*, and *Triticum aestivum*, respectively. Gray lines (background) indicate collinear blocks within barley and other plant genomes; red lines highlight syntenic HvTALE gene pairs.

To explore evolutionary clues of TALE members among barley (*H. vulgare*) and other species, three dicots (*A. thaliana*, *G. max*, and *P. trichocarpa*) and three monocots (*O. sativa*, *Z. mays*, and *T. aestivum*) were used for syntenic analyses (**Supplementary Table 6B**). In total, 3, 2, 5, 15, 56, and 14 HvTALE orthologous genes were identified for these species (**Supplementary Figure 3**). Output results were integrated into the comparative syntenic schematics in **Figure 4**. Two barley TALE members (*HvBELL2* and *HvBELL3*) have collinear pairs with five species. Eight barley TALE members have collinear pairs with three monocot species. Thus, barley has more collinear pairs with the monocots *O. sativa*, *Z. mays*, and *T. aestivum*.

**Figure 5:**
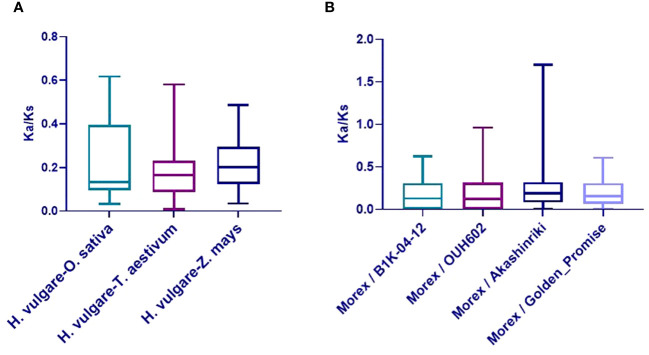
Boxplot of non-synonymous/synonymous substitutions (Ka/Ks) ratios in orthologous gene pairs. **(A)** Ka/Ks values of TALE orthologous gene pairs among barley (*Hordeum vulgare*) and three species (*Oryza sativa*, *Zea mays*, and *Triticum aestivum*). **(B)** Ka/Ks values of barley TALE homologous gene pairs among Morex and four barley accessions (Akashinriki, Golden_Promise, B1K-04–12, and OUH602).

The Ka/Ks ratios of TALE orthologous gene pairs between barley and the six species were also calculated to uncover the evolutionary constraints on the TALE superfamily (**Supplementary Table 6B**). All orthologous TALE gene pairs show Ka/Ks < 1, indicating that the barley TALE superfamily has undergone purifying selective pressure in monocotyledons (Han et al., 2022). Obtained Ka/Ks ratio values among barley and three monocot species are shown in **Figure 5A**.

Variations in TALE genes among five barley accessions (Morex, Akashinriki, Golden Promise, B1K-04–12, and OUH602) were investigated. Approximately 105 TALE family genes were identified from three cultivated and two wild accessions, with 21 genes in each (**Supplementary Table 3**). Ka/Ks values of barley TALE homologous gene pairs among Morex and four barley accessions were calculated to study selection pressure on the TALE superfamily during barley domestication (**Supplementary Figure 3**, **Supplementary Table 7**). Excepting *BELL4* (Ka/Ks = 1.70) between Morex and Akashinriki, the Ka/Ks values were all <1, indicating that TALE superfamily genes are continuously evolving through purification selection. It is possible that *BELL4* in Akashinriki (cultivated barley) had great artificial variation. Ka/Ks values are depicted in **Figure 5B**.

### 3.5 Interaction network and protein interaction analysis of HvTALE proteins

**Figure 6:**
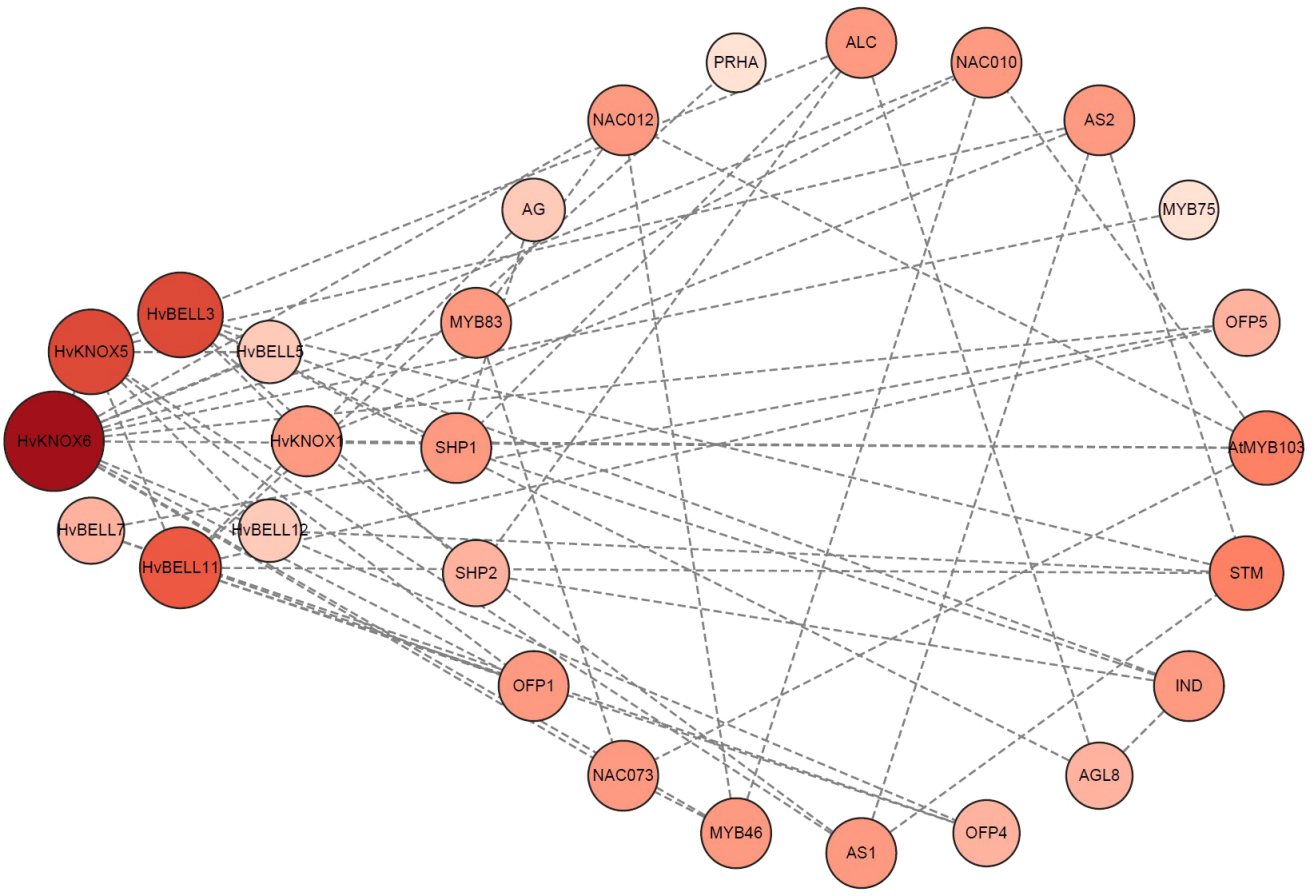
Predicted functional interaction networks of barley TALE proteins. The eight barley genes identified and marked in red are orthologs mapped using *Arabidopsis* genes. Medium confidence (0.400); 20 interactors.

Protein–protein interactions are central mediators in biological processes. Clarifying interaction relationships among HvTALE proteins is important to characterize their potential functions and regulatory pathways. A protein interaction network among HvTALE proteins prepared using STRING software predicted eight HvTALE family members to interact with other proteins (**Supplementary Table 8**, **Figure 6**). Excepting HvBELL7, seven HvTALE proteins could interact with other HvTALE members. HvKNOX5 was central to the interaction network and interacted strongly with five HvTALE members, including one KNOX subfamily member (HvKNOX6) and four BELL subfamily members (HvBELL3, HvBELL5, HvBELL11, and HvBELL12). Additionally, the BELL subfamily member (HvBELL5) may interact with KNOX subfamily member HvKNOX6, and HvBELL3 may interact with HvKNOX1. This is consistent with previous studies to form KNOX–BELL heterodimer proteins.

**Figure 7:**
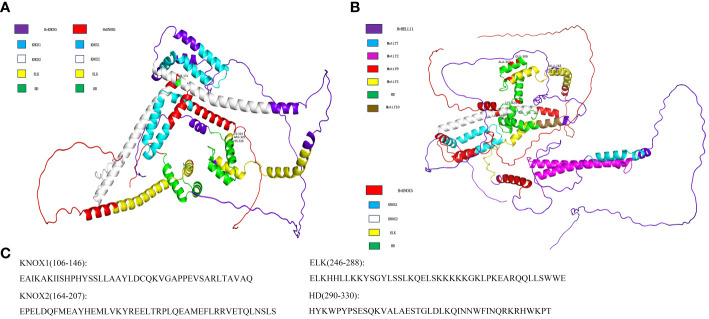
The 3D structure modeling of HvKNOX5–HvKNOX6 and HvKNOX5–HvBELL11 complexes. **(A)** HvKNOX5–HvKNOX6 complex with a hydrogen bonding site (Tyr174) on the KNOX2 domain of the HvKNOX5 protein and three consecutive bonding sites (Lys324, Arg325, and His326) on the C-terminal side of the HD domain of the HvKNOX5 protein. **(B)** HvKNOX5–HvBELL11 complex with a hydrogen bonding site (Leu189) on the KNOX2 domain of the HvKNOX5 protein, three hydrogen bonding sites (Ala303, Glu306, and Lys324) on the HD domain of the HvKNOX5 protein and two hydrogen bonding sites (Glu265 and Lys269) on the ELK domain of the HvKNOX5 protein. **(C)** KNOX1, KNOX2, ELK, and HD domain sequences of HvKNOX5.

On the basis of the HvTALE family interaction network, we selected HvKNOX6–HvKNOX5 and HvBELL11–HvKNOX5 protein interaction and simulated the spatial structure of their protein complexes. According to 3D structure modeling (**Figure 7**), HvKNOX5 and HvKNOX6 interact with each other through 11 hydrogen bonds and two salt bridges (**Supplementary Figure 5A**). The 3D modeling of the HvKNOX5–HvKNOX6 complex has a confidence score of 0.9775 (**Supplementary Table 9**). In the HvKNOX5–HvKNOX6 complex, the KNOX2 domain of the HvKNOX5 protein has one bonding site (Tyr174), and the C-terminal side of the HD domain has three consecutive hydrogen-bonding sites (Lys324, Arg325, and His326). HvKNOX5 and HvBELL11 interact with each other through 15 hydrogen bonds and one salt bridge (**Supplementary Figure 5B**). The 3D modeling of the HvKNOX5–HvBELL11 complex has a confidence score of 0.9763 (**Supplementary Table 9**). In the HvKNOX5–HvBELL11 complex, in addition to one hydrogen bonding site (Leu189) on the KNOX2 domain, there is another (Lys324) on the C-terminal side of the HD domain; the HvKNOX5 protein has two hydrogen bonding sites (Glu265 and Lys269) on the ELK domain and two (Ala303 and Glu306) on HD domains. HvKNOX5–HvKNOX6 and HvKNOX5–HvBELL11 complexes are formed through a hydrogen bonding site on the KNOX2 domain and a hydrogen bonding site on the C-terminal of the HD domain, which may be essential for the interaction between HvKNOX5 and other TALE superfamily members.

### 3.6 *cis*-Element analyses of barley TALE genes

**Figure 8:**
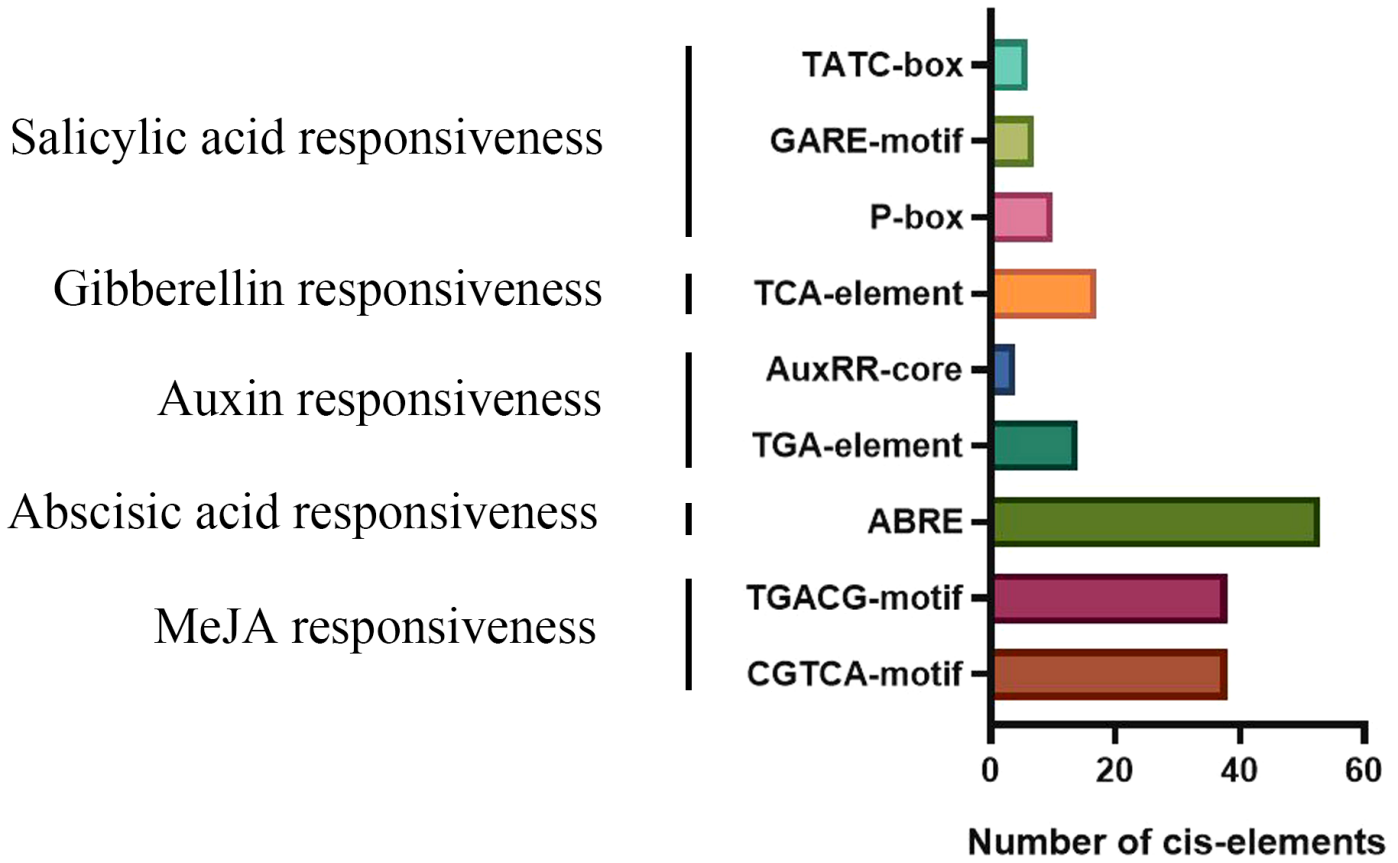
Number of plant hormone *cis*-elements detected in HvTALE genes.

*cis*-Elements are crucial for transcriptional control of gene expression (Wang et al., 2017). For *cis*-element studies, we extracted the HvTALE genes’ promoter region sequences, namely, the 2,000-bp upstream sequences from gene initiation codons (**Supplementary Table 10**). *cis*-Elements are proportionally displayed in **Supplementary Figure 6**. Interestingly, *cis*-elements with a wide distribution in gene promoter regions included light, hormone, defense, and stress responsiveness and anaerobic induction (**Supplementary Table 11**). The results also manifest that barley TALE members are probably linked to responses to plant hormones, such as abscisic acid (53), jasmonic acid methyl ester (76), salicylic acid (17), auxin (18), and gibberellin (23) (**Figure 8**). This means that the potential functions of HvTALE genes include responses to abiotic stressors and plant hormones and participation in various biological processes.

### 3.7 Expression profiling of HvTALEs in different barley tissues and responses to exogenous hormones

**Figure 9:**
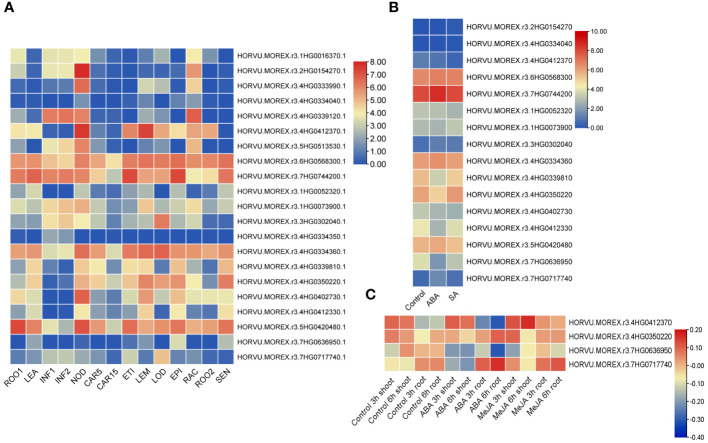
Expression heatmaps of *HvTALE* genes in different tissues and different responses to exogenous hormones. Expression heatmaps of *HvTALE* genes in **(A)** diverse tissues at different developmental stages [using profiles provided by the Leibniz Institute of Plant Genetics and Crop Plant Research (IPK)], **(B)** responses to ABA and SA [using Sequence Read Archive (SRA) samples], and **(C)** different responses to ABA and MeJA [using Gene Expression Omnibus (GEO) BioProject samples].

Expression patterns of barley TALE genes were analyzed by generating a tissue-specific expression heatmap (**Supplementary Table 1E**, **Figure 9**). Four TALE genes show very low or no expression, with log2(FPKM+1) < 1 for all developmental stages; others were expressed with log2(FPKM+1) > 1 in at least one organ. Of them, transcription levels of *HvKNOX8* (*HORVU.MOREX.r3.6HG0568300.1*), *HvKNOX9* (*HORVU.MOREX.r3.7HG0744200.1*), *HvBELL5* (*HORVU.MOREX.r3.4HG0334360.1*), and *HvBELL10* (*HORVU.MOREX.r3.5HG0420480.1*) are high at all stages. Most HvTALE genes are expressed more strongly in the third internode of tillers than in the other organs. The expression of *HvKNOX6* (*HORVU.MOREX.r3.4HG0412370.1*) was abundant in the third internode of tillers and lemma.

We also analyzed expression profiles of barley HvTALE genes responding to plant hormones based on public RNA-seq datasets. Based on mapping all reads from SRA and GEO datasets to the Morex v3 genome, we found that there were 16 reads in SRA datasets (ABA and SA treats) (**Supplementary Table 1A**) while only four reads in the GEO datasets (ABA and MeJA treats) (**Supplementary Table 1C**) mapping to TALE genes from the Morex v3 genome. From the overall trend, under ABA and SA stress, KNOX family members were basically unchanged compared with the control in 14-d leaves after seedling, and most BELL family members [e.g., *HvBELL6* (*HORVU.MOREX.r3.4HG0339810*), *HvBELL9* (*HORVU.MOREX.r3.4HG0412330*), and *HvBELL11* (*HORVU.MOREX.r3.7HG0636950*)] were slightly downregulated (**Figure 9**). GEO sample data reveal that four genes in the development of shoots and roots exhibit expression changes under MeJA treatments (**Figure 9**), of which *HvBELL12* (*HORVU.MOREX.r3.7HG0717740*), *HvBELL7* (*HORVU.MOREX.r3.4HG0350220*), and *HvKNOX6* (*HORVU.MOREX.r3.4HG0412370*) were upregulated compared with the control and *HvBELL11* (*HORVU.MOREX.r3.7HG0636950*) was downregulated. Under ABA stress, *HvKNOX6* (*HORVU.MOREX.r3.4HG0412370*) was upregulated in the shoot compared with the control, and three BELL family members [*HvBELL12* (*HORVU.MOREX.r3.7HG0717740*), *HvBELL7* (*HORVU.MOREX.r3.4HG0350220*), and *HvBELL11* (*HORVU.MOREX.r3.7HG0636950*)] were downregulated. Additionally, *HvBELL12* (*HORVU.MOREX.r3.7HG0717740*) and *HvBELL7* (*HORVU.MOREX.r3.4HG0350220*) increased significantly in the roots in ABA treatments compared with the control, and *HvKNOX6* (*HORVU.MOREX.r3.4HG0412370*) and *HvBELL11* (*HORVU.MOREX.r3.7HG0636950*) genes showed much lower expressions.

### 3.8 Expression patterns of *HvTALE*s responding to exogenous hormones in seedling leaves using quantitative RT-PCR

**Figure 10:**
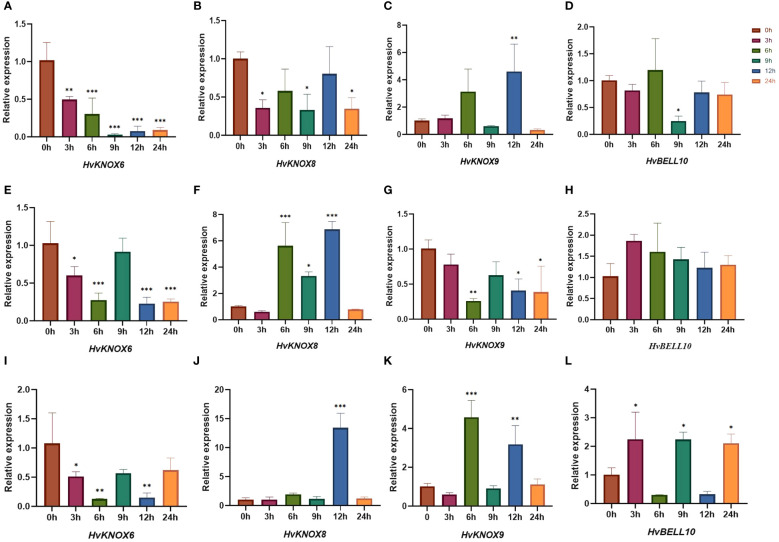
*HvTALE* gene expression patterns responding to exogenous hormones in seedling leaves. The expression patterns of **(A)** *HvKNOX6*, **(B)** *HvKNOX8*, **(C)** *HvKNOX9*, and **(D)** *HvBELL10* in response to the ABA treatment. The expression patterns of **(E)** *HvKNOX6*, **(F)** *HvKNOX8*, **(G)** *HvKNOX9*, and **(H)** *HvBELL10* in response to the GA3 treatment. The expression patterns of **(I)** *HvKNOX6*, **(J)** *HvKNOX8*, **(K)** *HvKNOX9*, and **(L)** *HvBELL10* in response to the 6-BA treatment. Asterisks indicate significant differences from controls (0-h sample points) and others (3 h, 6 h, 9 h, 12 h, and 24 h): *p < 0.05, **p < 0.01, ***p < 0.001, Student’s t-test.

Based on previous studies (Zhang et al., 2021; Han et al., 2022), we used qRT-PCR to detect expression levels under ABA, GA3, and 6-BA stress for four TALE genes that were relatively highly expressed in each tissue (**Figure 10**; **Supplementary Table 12**). When treated with ABA, the expression levels of *HvKNOX6* and *HvNOX8* decreased more significantly than those of their controls (the 0-h sample point). Compared with that of the control groups, the expression level of *HvKNOX9* was downregulated at 3-h, 6-h, 9-h, and 24-h sample points but upregulated at 12 h. The expression level of *HvBELL10* did not differ significantly from controls at 3 h, 6 h, 12 h, and 24 h but was significantly decreased at 9 h. For GA3 treatments, the expression levels of *HvKNOX6* and *HvNOX9* trended downward compared with controls, while those of *HvBELL10* trended upward at all sample points. At 6 h, 9 h, and 12 h, the expression levels of *HvNOX8* increased more significantly than control values. When treated with 6-BA, the expression level of *HvKNOX6* also decreased more significantly than controls. The expression levels of *HvNOX8* increased more significantly compared with controls at 12 h. The expression levels of *HvNOX9* did not differ significantly from controls at 3 h, 9 h, and 24 h but increased significantly compared with its controls at 6 h and 12 h. The expression levels of *HvBELL10* at 3 h, 9 h, and 24 h were significantly higher than for controls. For treatments of exogenous ABA, GA3, and 6-BA, three KNOX II genes showed relatively complicated expression patterns at 3 h, 6 h, 9 h, 12 h, and 24 h, possibly indicating that each gene had a different regulating function in barley (Matsumoto et al., 2014; Hartmann et al., 2022).

## 4 Discussion

### 4.1 Identification and evolutionary relationships of TALE gene family in barley

Members of the TALE superfamily are essential for regulating plant development, growth, and hormone responses. We comprehensively investigated the HvTALE gene family and provided perspectives into their biological functions. The number of TALE gene family members varies between taxa: *Arabidopsis* (22), poplar (35), soybean (68), and wheat (70) (Hamant and Pautot, 2010; Zhao et al., 2019; Wang et al., 2021; Han et al., 2022). In barley, the total number of TALE members identified (21) is close to that in diploid *Arabidopsis* but below that in polyploid plants (triploid poplar, tetraploid soybean, and hexaploid wheat). Therefore, we speculate that numbers of TALE superfamily genes are associated with species ploidy levels.

To explore the phylogeny and evolutionary relationships among TALE family genes, a phylogenetic tree comprising five species (four dicotyledons and one monocotyledon) was constructed. With reference to the classification in *Arabidopsis*, we divided barley TALE gene family members into KNOX and BELL-like subfamilies. In barley, KNOX subfamily members are further divided into class I KNOX and class II KNOX. The BELL-like subfamily has not been previously systematically classified. In terms of the conserved domains of HvTALE members, all HvTALE members contained HD domains. The BELL-like subfamily members harbored POX domains, but the KNOX subfamily had only KNOX1, KNOX2, and ELK domains (Müller et al., 2001; Cole et al., 2006). Our classification results are supported by the specific domains or motif combinations found in each HvTALE subfamily and hereditary class. Introns are essential for both evolution and the production of new gene family members (Roy and Penny, 2007). Investigating the evolution of HvTALE genes may be aided by understanding the intron distribution patterns of these genes. HvTALE gene structures are distinct in different subfamilies. All genes in the KNOX subfamily contain five exons, and most BEL1-like subfamily members contain four exons (except *HvBELL4* and *HvBELL7*). Members of HvTALE may exhibit distinctive divergences and consistencies that indicate their functional differences and comparability, and members of the same TALE branch may perform comparable biological functions.

Segmental and tandem gene replication plays a driving role in the evolutionary expansion of plant gene families, and low-tandem, high segmental duplication classes are involved in various enzymatic functions (Cannon et al., 2004; Zhang et al., 2018). Gene duplication analysis revealed that most *HvTALE* genes had originated from segmental duplications, further identifying the crucial role that these segmental duplications play in barley TALE gene family expansion. Syntenic gene pairs among species may be useful for evolutionary research on the gene family (Han et al., 2022). To further investigate evolutionary clues from *HvTALE* genes, three dicotyledons and three monocotyledons were recruited for syntenic analysis (**Figure 4**). More *HvTALE* orthologous genes were detected in monocotyledons than in dicotyledons, and barley and wheat had the most syntenic pairs. This indicates that syntenies among TALE genes may parallel the evolutionary divergence of species. Important homologous pairs are often highly conserved, and they may exist before taxonomic differentiation (Xie et al., 2018; Wang et al., 2020). We report HvBELL2 and HvBELL3 to show homologous pairing in dicotyledonous and monocotyledonous, indicating that this conserved pairing may have existed before monocotyledons separated from dicotyledons.

Selective pressure at the protein level is usually measured by the non-synonymous/synonymous rate ratio (Ka/Ks), with Ka/Ks < 1, Ka/Ks = 1, and Ka/Ks > 1 indicating purifying (or negative) selection, neutral evolution, and diversifying (or positive) selection, respectively (Anisimova et al., 2001). Except for the *BELL4* Ka/Ks value (1.70) between the Morex and Akashinriki varieties of barley, Ka/Ks values were all <1, indicating that the barley TALE superfamily member had undergone a strong purifying selection. The Ka/Ks value of *BELL4* between the Morex and Akashinriki varieties was >1, possibly because *BELL4* of Akashinriki had been subject to abnormal artificial mutation.

### 4.2 Prediction and 3D structure modeling of barley TALE proteins

The protein interactions between members of the TALE gene family are widespread in plants. For example, the interaction between the KNAT3 protein in *Arabidopsis* and the BLH1 protein can influence how plants respond to ABA and may also indirectly influence how resilient plants are to adversity (Bhatt et al., 2004; Kim et al., 2013). Certain GhBEL1-like proteins in cotton interact with GhKNAT7 homologs to influence the network responsible for the formation of fiber SCWs (Ma et al., 2019). In protein interactions, some domains or patterns are crucial (Liu et al., 1999). The KNOX1 and KNOX2 domains comprise the MEINOX region. The KNOX1 domain is crucial for reducing the expression of the target gene, while the functionally essential KNOX2 domain is thought to be necessary for dimerization (Nagasaki et al., 2001); even the KNOX2 domain alone can interact with the BELL protein (Liu et al., 2014). In *Arabidopsis*, the MEINOX domain in KNOX proteins mediated BEL1 and particular KNOX protein interactions to form heterodimers (Bellaoui et al., 2001). KNATM proteins that have lost their HD domains also use the MEINOX domain to selectively bind with *Arabidopsis* BELL proteins (Magnani and Hake, 2008). The HD domain of MdKNOX15 in apple can also interact with MdBLH1 through yeast double hybridization (Jia et al., 2021a).

How KNOX–KNOX and KNOX–BELL interact in the TALE gene family may vary from species to species. Compared with KNOX proteins, there has been less research performed on BELL proteins, which contain an HD and MEINOX interacting domain composed of POX domains (Müller et al., 2001; Cole et al., 2006). In this study, through interaction network analysis, we report that barley HvKNOX5 has strong interactions with five HvTALE members (HvKNX6, HvBELL3, HvBELL5, HvBELL11, and HvBELL12). Accordingly, we chose HvKNOX5–HvKNOX6 and HvKNOX5–HvBELL11 complexes for protein interaction studies. In the HvKNOX5–HvKNOX6 complex, the KNOX2 domain of the HvKNOX5 protein has one hydrogen bonding site (Tyr174), and the HD domain has three consecutive hydrogen bonding sites (Lys324, Arg325, and His326). In the HvKNOX5–HvBELL11 complex, HvKNOX5 has two hydrogen bonding sites (Glu265 and Lys269) on the ELK domain, one hydrogen bonding site (Leu189) on the KNOX2 domain, and three hydrogen bonding sites (Ala303, Glu306, and Lys324) on the HD domain. The C-terminal of the MEINOX domain of KNOX3 that is the former name of HvKNOX5 in barley is necessary for KNOX–KNOX and KNOX–BELL complex interactions (Müller et al., 2001). We report the barley HvKNOX5 protein to have a hydrogen binding site on the KNOX2 domain, regardless of whether it binds to HvKNOX6 or HvBELL11.

### 4.3 The roles of HvTALEs in phytohormone responses

Excepting in the third internode of tillers and developing grains 5 d and 15 d after anthesis, the expression level of KNOX I genes was relatively low in different barley developmental stages, consistent with results reporting KNOX I member expression in meristems (Hake et al., 2004; Hay and Tsiantis, 2010; Qi and Zheng, 2013). Additionally, KNOX genes control the metabolic processes and signaling pathways linked to several hormones, such as cytokinin, auxin, and gibberellin (Tsuda and Hake, 2015). Exogenous ethylene treatment could promote the downregulation of *Bkn3*, which has a 305-bp insertion sequence in the fourth intron of *HvKNOX5* and change the phenotype of the barley hooded mutation (Osnato et al., 2010). In apple, *MdKNOX19* (a class II KNOX gene) was significantly upregulated when ABA was applied to its seeds, leaves, and fruits (Jia et al., 2021b). We report many *cis*-elements in the 2,000-bp promoter sequence to respond to hormones, indicating that most TALE superfamily members may be inducible by exogenous phytohormones. Excepting *HvKNOX9* at 6 h and 12 h, we report ABA to inhibit the expression of class II KNOX genes in barley leaves. From these SRA sample data, the expression of KNOX II family members under ABA stress is unchanged compared with control values. According to GEO sample data, the expression of the KNOX II family member *HvKNOX6* differs in shoots and roots under ABA stress. Accordingly, we conclude that barley KNOX II subfamily members may have different expressions in different growth periods, tissues, and organs in ABA treatments. Class KNOX I genes are TFs that help preserve meristem identity by inhibiting GA production and stimulating cytokinin synthesis (Hay and Tsiantis, 2010). Compared with controls, we report the expression of *HvKNOX6* and *HvKNOX9* to trend downward and for *HvKNOX8* to trend upward with GA3 treatment. Therefore, the expression of class II KNOX genes responds differently to GA3. In *Arabidopsis*, class KNOX I genes can be mediated by cytokinin to promote cell division and maintain meristem (Jasinski et al., 2005). Although we found no CK-responsive *cis*-elements in the promoter sequence of the barley KNOX family, qRT-PCR results indicate that cytokinin (6-BA) also has a regulatory effect on class II KNOX genes in barley. Therefore, we assume that CK regulates the expression of KNOX II genes by regulating other genes.

Few studies have examined interactions between BELL genes and hormones. We report *HvBELL11* to be downregulated compared with control values under ABA, SA, and MeJA stress. From the interaction network among HvTALE proteins, HvBELL11 may interact with HvKNOX5. Given that HvKNOX5 plays an important regulatory role in the formation of barley awns (Osnato et al., 2010), the regulatory relationship between them warrants study in specific barley tissues (such as awns).

## Data availability statement

The original contributions presented in the study are included in the article/**Supplementary Material**. Further inquiries can be directed to the corresponding author.

## Author contributions

T-JL: Conceptualization, Data curation, Investigation, Writing – original draft, Writing – review & editing. TH: Formal analysis, Investigation, Software, Writing – original draft. H-YX: Funding acquisition, Writing – original draft, Writing – review & editing. J-CD: Data curation, Investigation, Writing – original draft. J-ZM: Data curation, Investigation, Writing – original draft. M-YD: Data curation, Investigation, Writing – original draft. R-JD: Conceptualization, Funding acquisition, Investigation, Writing – original draft, Writing – review & editing.
