# Supplementary material for: Genome-wide identification, characterization, and evolutionary analysis of the barley TALE gene family and its expression profiles in response to exogenous hormones
Source: Front Plant Sci. 2024 Jun 27;15:1421702. doi: 10.3389/fpls.2024.1421702 (PMC11236544; doi:10.3389/fpls.2024.1421702)
Supplement: Supplementary file 13 [file DataSheet_1.docx]

Supplementary Material

**Genome-wide identification, characterization, and evolutionary analysis of barley TALE genes and their expression profiles in response to exogenous hormones**

**Tian-jiang Liao^1,2^, Tao Huang^1^, Hui-yan Xiong^2^, Jie-cuo Duo^1^, Jian-zhi Ma^1^, Ming-yang Du^1^, Rui-jun Duan^1^****^,2*^**

1 College of eco-environmental engineering, Qinghai University, Xining 810016, Qinghai, China

2 College of Agriculture and Animal Husbandry, Qinghai University, Xining 810016, Qinghai, China.

* **Correspondence:**Corresponding Author: ruijunduan@163.com

## Supplementary Figures

**
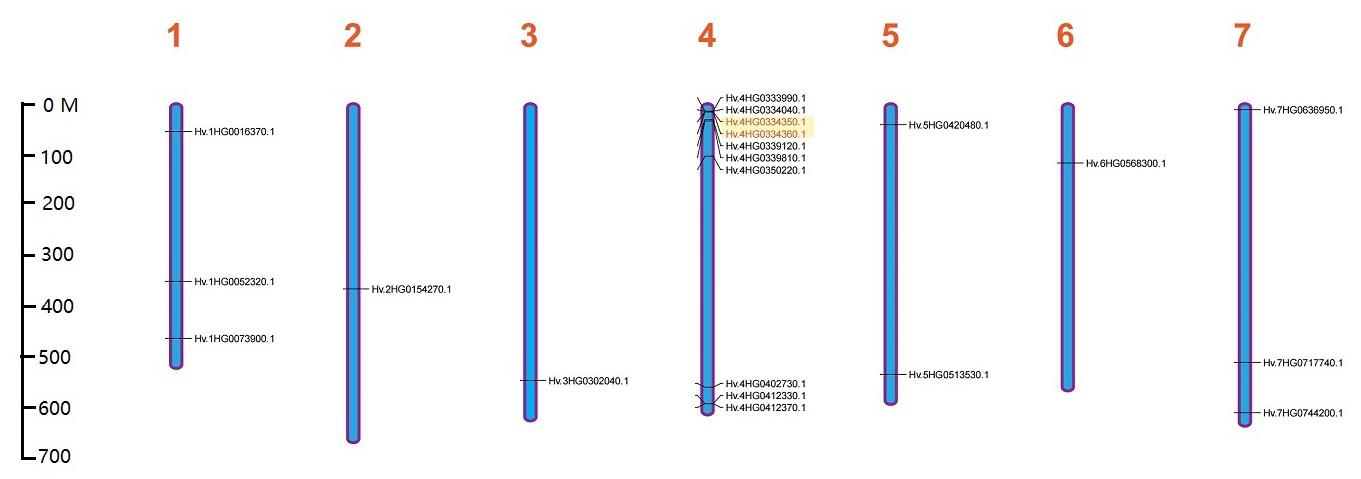
**

**Supplementary Figure S1.** Chromosomal distributions of HvTALE genes. Chromosomal names were placed on the top. The scale on the left is in megabases. HvTALE gene names on chromosomes were placed on the right. Besides, the tandemly duplicated HvTALE gene pair was linked by orange.


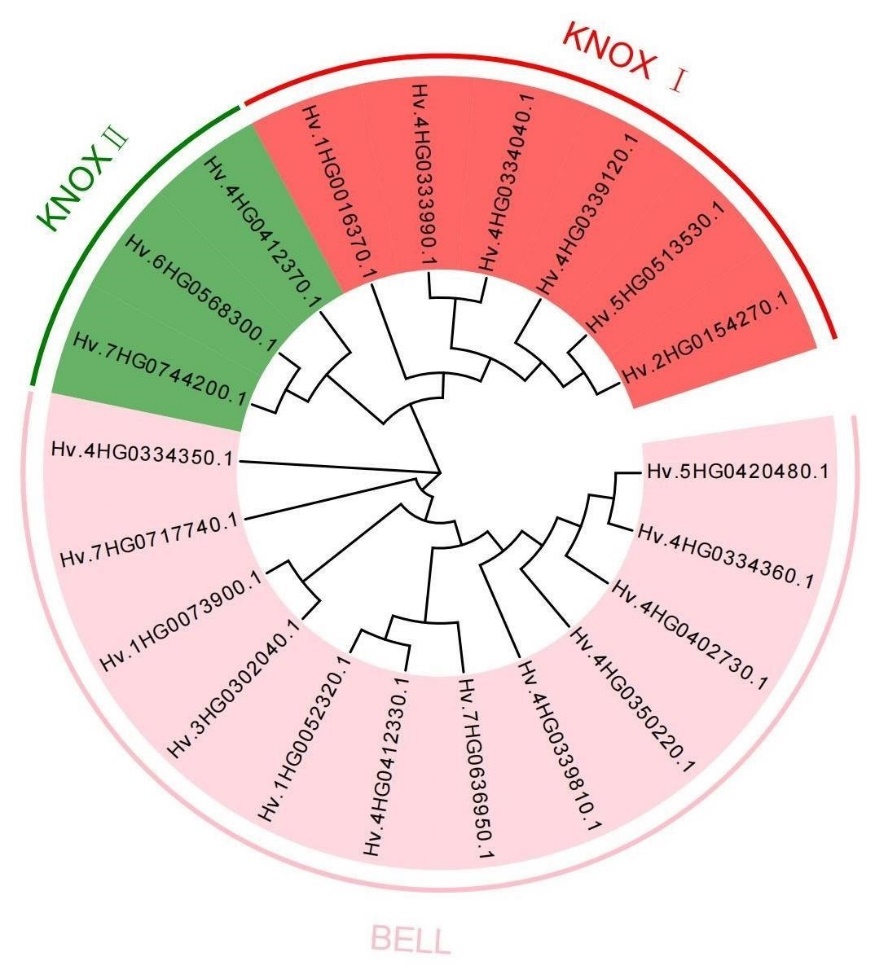


**Supplementary Figure S2.** Unrooted neighbor-joining tree constructed from barley TALE proteins. Different subclass genes are distinguished by different colors, KNOX Ⅰ: red, KNOX Ⅱ: green, and BELL: pink.


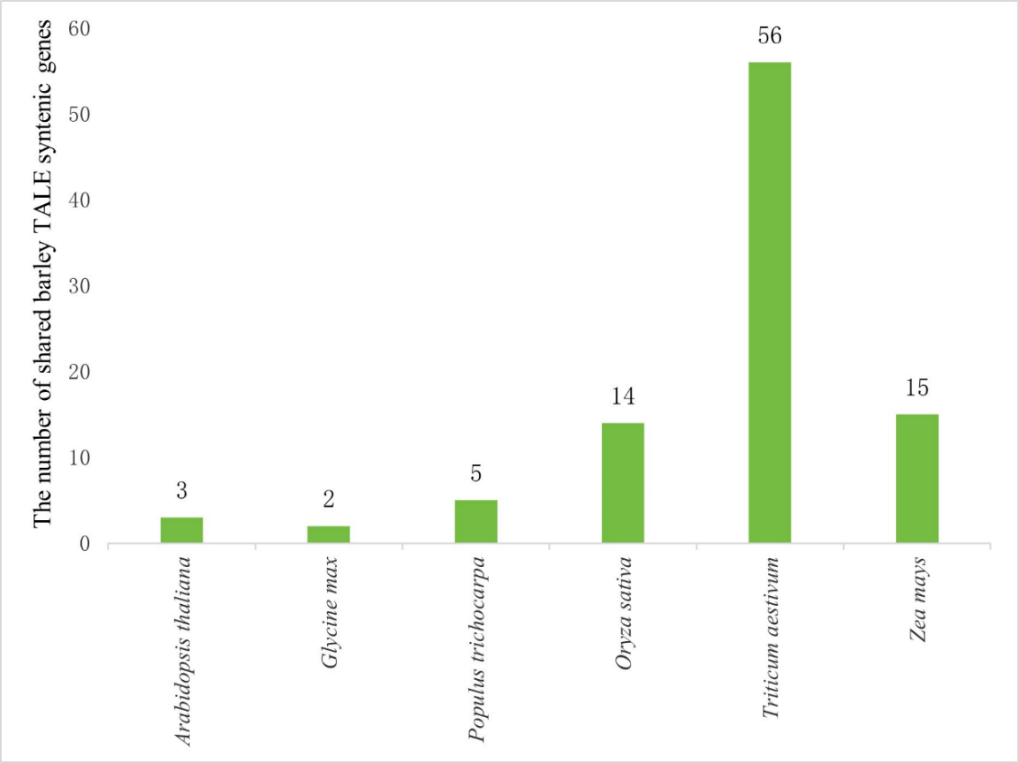


**Supplementary Figure S3.** HvTALE orthologous genes between *Hordeum vulgare* and *Arabidopsis thaliana*, *Glycine max*, *Populus trichocarpa*, *Oryza sativa*, *Triticum aestivum*, *Zea mays*, respectively*.*


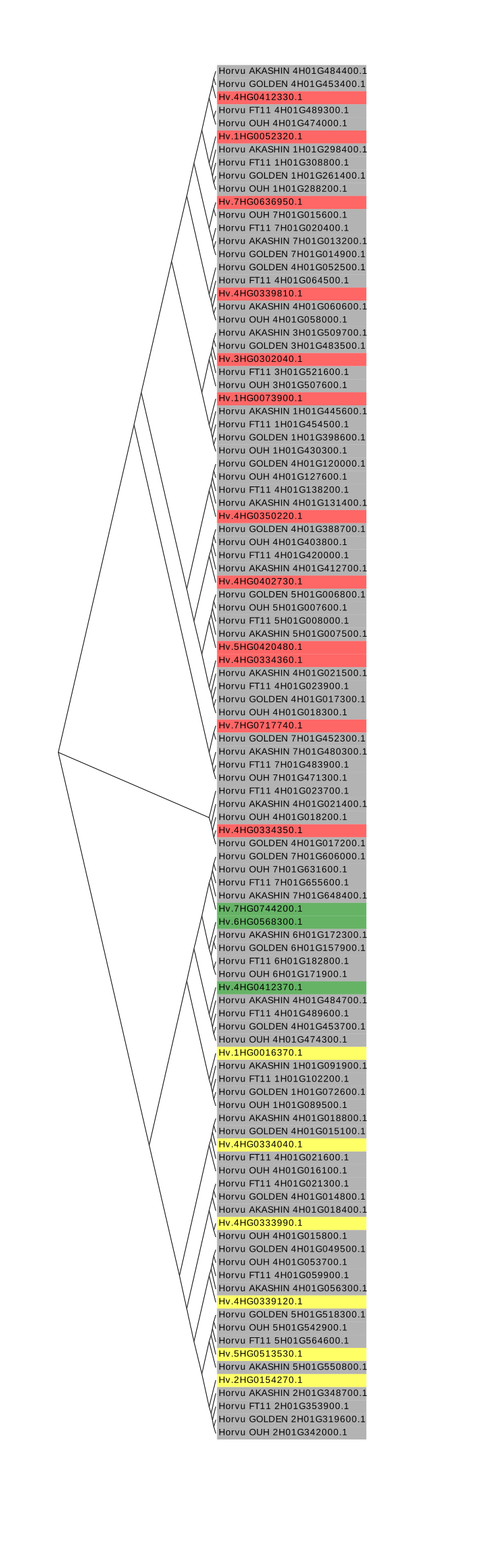


**Supplementary Figure S4.** Phylogeny of TALE family proteins between wild (OUH602, B1K-04-12) and cultivated barley (Morex, Akashinriki, Golden Promise). KNOX Ⅰ genes of Morex are shown in yellow, KNOX Ⅱ genes of Morex are shown in green, and BELL genes of Morex are shown in red. The neighbor-joining tree was constructed with Muscle in MEGA7 software based on an alignment of full-length amino acid sequences. Bootstrap probabilities are shown at each branch node.


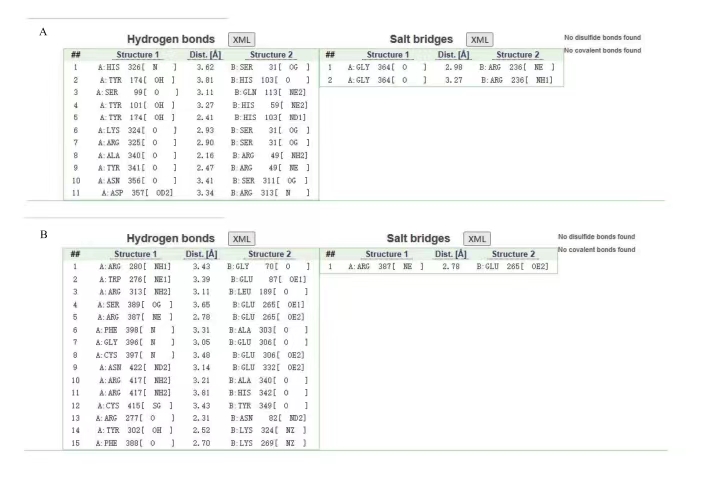


**Supplementary Figure S5.** Hydrogen bonds and salt bridge of two TALE protein complexes in barley. (A) Eleven hydrogen bonds and two salt bridges of HvKNOX5–HvKNOX6 complex. Structure 1 is the structure modeling of HvKNOX5; Structure 2 is the structure modeling of HvKNOX6. (B) Fifteen hydrogen bonds and one salt bridge of HvKNOX5–HvBELL11complex. Structure 1 is the structure modeling of HvBELL11; Structure 2 is the structure modeling of HvKNOX5.


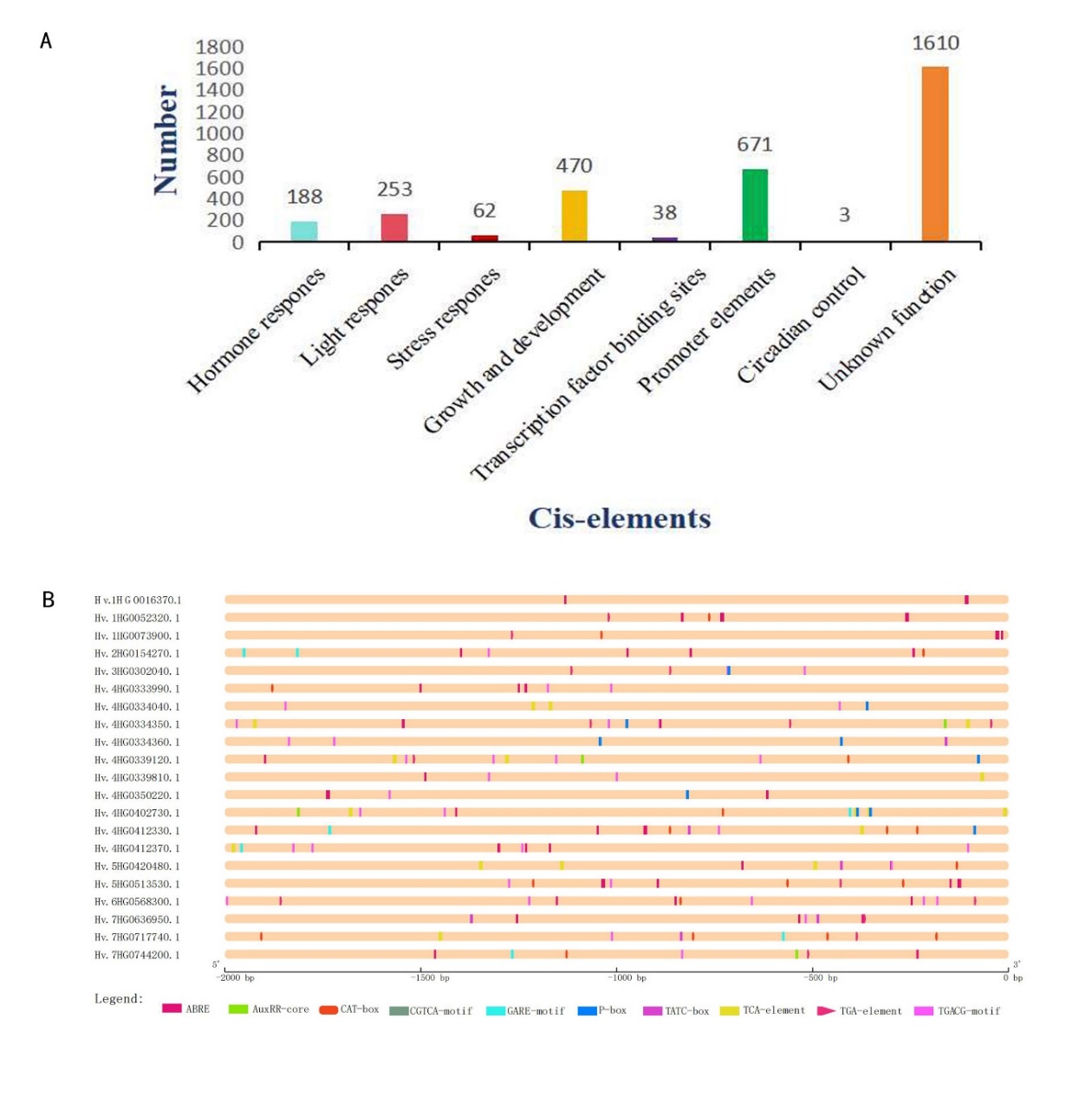


**Supplementary Figure S6.** Total number of cis-elements detected in barley TALE genes. (A) Statistics of cis-elements found in barley TALE genes, such as light responsive, hormone responsive, growth and development, stress responsive, transcription factor binding sites, and unknown function. (B) Cis-elements responding to plant hormones of barley TALE genes. Number of cis-elements per TALE genes heatmap; different colors indicate the number of cis-elements. Different types of cis-elements are shown in different colors.
